# A new neuropsychological tool for simultaneous reading and executive functions assessment: initial psychometric properties

**DOI:** 10.3389/fpsyg.2024.1399388

**Published:** 2024-09-23

**Authors:** Vinícius Figueiredo de Oliveira, Jéssica Vial-Martins, André Luiz de Carvalho Braule Pinto, Rochele Paz Fonseca, Leandro Fernandes Malloy-Diniz

**Affiliations:** ^1^Laboratory of Medical Psychology and Neuropsychology, Department of Mental Health, Faculty of Medicine, Federal University of Minas Gerais, Belo Horizonte, Brazil; ^2^Amazonas Psychological Assessment Laboratory, Faculty of Psychology, Federal University of Amazonas, Manaus, Brazil; ^3^Experimental and School Neuropsychology, Faculty of Medicine, Federal University of Minas Gerais, Belo Horizonte, Brazil

**Keywords:** reading, reading comprehension, executive functions, psychometric validation, inhibitory control, cognitive flexibility, working memory, neuropsychological assessment

## Abstract

**Introduction:**

The development of reading and complex executive functions is fundamental for achieving social, academic, and professional success. So far, there is no single neuropsychological instrument that comprehensively assesses the domains of inhibitory control, cognitive flexibility, working memory, and reading comprehension. To assess executive functions related to reading, the “Assessment of Reading and Executive Functions” (AREF) was developed. In this study, we show initial evidence of validity and reliability for four subtests - Graphophonological-Semantic Flexibility, Inhibitory Control, Flexibility, and Working Memory.

**Methods:**

A total of 93 students from 4th to 9th grade, aged 8-14, in public (*n* = 61) and private (*n* = 32) schools were evaluated. Tasks from the AREF instrument, as well as measures of reading comprehension, inhibitory control, cognitive flexibility, working memory, and intelligence, were administered. Correlations between AREF scores and the other measures were performed to assess external construct validity. Performance differences between school groups on AREF subtests were analyzed using ANOVA, t-test, and Mann-Whitney tests, and the internal consistency of the instrument’s tasks was evaluated using Cronbach’s alpha coefficient.

**Results:**

The scores of the AREF subtests demonstrated significant positive correlations with reading measures (ranging from 0.339 to 0.367) and executive functions (ranging from 0.209 to 0.396). Significant differences were found in the performance of some AREF tasks when comparing individuals from public and private schools, as well as between 4th and 5th graders compared to students in higher grades. The internal consistency of the tasks was low for Graphophonological-Semantic Flexibility (Cronbach’s α = 0.566), moderate for Inhibitory Control and Flexibility (Cronbach’s α = 0.768), and high for Working Memory (Cronbach’s α = 0.881).

**Discussion:**

The results provide initial evidence of construct validity and reliability for the AREF subtests. It is expected that this new neuropsychological test will contribute to the assessment of reading skills and executive functions, assisting in guiding clinical and educational interventions for individuals with and without neurodevelopmental disorders.

## 1 Introduction

The development of reading and executive functions represents a central area of interest in cognitive research, given its intricate complexity and broad implications for cognitive development (Peng and Kievit, 2020; Burgess and Cutting, 2023). Competence in reading not only stands as a crucial element for academic and professional success but is also imperative for full integration into society (Rabiner et al., 2016; OECD, 2023). However, the acquisition of reading skills is a multifaceted and challenging process, extending beyond mere word decoding to demand equally meaningful comprehension of textual content (Dehaene, 2009; Fonseca et al., 2020).

From a theoretical standpoint, the dual-route cognitive model has often been employed to describe the decoding process in reading, emphasizing the interaction between orthographic-visual analysis and the lexical and phonological routes (Coltheart et al., 2001). However, while this process is fundamental, it proves insufficient to achieve a substantial level of reading proficiency (Kendeou et al., 2014). Therefore, other cognitive processes are also implicated in reading, allowing us to transcend the scope of mere lexical decoding.

One proposal seeking to explain reading comprehension by incorporating decoding into its model is the Simple View of Reading (Gough and Tunmer, 1986). According to this hypothesis, reading comprehension results from Decoding X Linguistic Comprehension, illustrating that reading requires the contribution of both variables for its effectiveness. It is widely accepted that the ability to decode text constitutes a fundamental requirement for comprehension (Perfetti and Hogaboam, 1975). Nevertheless, the Simple View of Reading appears to solely focus on bottom-up processes involved in the activity, rather than presenting a suggestion that includes metacognitive abilities for the reader to assimilate the content of the text (Spencer et al., 2020).

One of the cognitive domains most closely related to effective processing of reading and textual comprehension is executive functions (EFs) (Gonçalves et al., 2017; Follmer, 2018). Executive functions comprise a set of high-level cognitive processes that enable flexible adaptation to diverse contexts, suppression of inappropriate impulsive responses, and temporary maintenance of crucial information in a variety of situations (Diamond, 2013). They are responsible for the regulation and supervision of complex tasks involving planning, decision-making, and problem-solving (Diamond, 2013).

Although there is no consensus regarding the components of executive functions, Miyake et al. (2000) relied on psychometric data to assess the validity of the three-factor model. Following the administration of executive function tests in a sample, confirmatory factor analysis was conducted, which supported the three components: shifting, updating (monitoring and maintaining information in working memory), and inhibition (inhibition of dominant or prepotent responses). The results indicated that, although moderately correlated, the factors are distinct constructs. Diamond (2013) maintains that the three-factor model has been supported in numerous neuropsychological studies, wherein working memory, inhibitory control, and cognitive flexibility comprise the core functions. Working memory refers to the ability to temporarily retain and manipulate information. Inhibitory control consists of the ability to restrain automatic or ongoing behaviors and suppress irrelevant stimuli. Cognitive flexibility, on the other hand, enables adaptation to changes in rules or environmental stimuli, resulting in behavioral adjustments.

Executive functions begin their development in childhood and continue to develop during adolescence, reaching maturity in adulthood (Romine and Reynolds, 2005). Despite this continuous growth, development is not linear, as skills may show more pronounced improvements depending on the period of life and the construct being analyzed (Huizinga et al., 2006). For example, from early childhood, rudimentary behaviors of inhibition, information manipulation, and flexibility are already observable (Capilla et al., 2004). Childhood, in particular, is a crucial period for the rapid development of executive functions, with significant improvement between the ages of 5 and 7, followed by a moderate effect between 8 and 15 years, and a lesser effect between 15 and 17 years (Best et al., 2011).

With the onset of schooling, the development of executive functions occurs simultaneously with the enhancement of reading ability. In the early school years, students learn the basic principles of word decoding and, in subsequent years, automate this skill to eventually comprehend the texts they read (Verhoeven and Perfetti, 2011). However, it is unclear whether reading and executive functions develop independently, without one influencing the trajectory of the other, or bidirectionally, where one ability affects the other through mutually beneficial interactions (Peng and Kievit, 2020). For example, a meta-analysis investigating the relationship between working memory and reading in individuals aged 4 to 80 years demonstrated that this relationship increases with age, suggesting a bidirectional effect between these skills (Peng et al., 2018). However, in Follmer’s (2018) meta-analysis examining the relationship between executive functions and reading comprehension from ages 6 to adulthood, the relationship was more pronounced in children than in adults. Regardless of the type of relationship between the developmental trajectories of the two constructs, current evidence supports the hypothesis that executive functions are fundamental for competent reading comprehension processing, directly influencing the ability to extract accurate information from text, interpret meanings, and maintain attentional focus (Butterfuss and Kendeou, 2018).

For example, in the decoding of isolated words, executive functions play an important role in the simultaneous assimilation of their phonological, orthographic, and semantic information (Cartwright, 2007; Varghese and Shanbal, 2024). Similarly, to achieve text comprehension, cognitive flexibility, inhibitory control, and working memory operate in particular ways. Cognitive flexibility, for example, is related to the ability to modify strategies applied to text reading, as it involves a process that requires planning (Latzman et al., 2010). Inhibitory control, in turn, plays a crucial role in suppressing previously acquired ineffective reading habits (Kieffer et al., 2013) and in inhibiting irrelevant information for text comprehension (Butterfuss and Kendeou, 2018). Finally, working memory plays a recognized role in text comprehension as it supports the retention, manipulation, and association of ideas read (Follmer, 2018). The study by Spencer et al. (2020) emphasized that proficient reading comprehension, as well as the ability to make inferences, requires the reader to manipulate information from multiple sources, including their prior knowledge. These processes demand the use of working memory.

Currently, there are several useful paradigms for assessing reading comprehension, such as those based on response formats like Cloze, Multiple Choice, Open Ended, Retell, and Picture Selection (Collins and Lindström, 2021). Similarly, there is a variety of instruments focused on measuring executive functions, such as the Card Sorting Paradigm (e.g., Wisconsin Card Sorting Test), Continuous Performance Test, Go/No-Go, Hayling and Brixton, Span, and Stroop, among others (Nyongesa et al., 2019). However, there is no instrument that utilizes the interaction between these two constructs to develop a paradigm allowing their simultaneous evaluation, for example, using words and texts to identify both reading skills and executive functions. This goal could be achieved through the application of reading tasks where executive demand progressively increases, so that accurate performance depends on both the recruitment of executive functions and reading ability. The absence of such a tool implies a missed opportunity to assess both constructs in a single battery of tasks, which could lead to greater practicality and efficiency in clinical and educational contexts, as well as differentiated analyses compared to existing paradigms.

In this regard, the development and validation of an assessment battery for the components of executive functions and reading comprehension emerge as a valuable strategy to identify students with deficits in these processes. The AREF - Assessment of Reading and Executive Functions (ALEFE - Avaliação da Leitura e das Funções Executivas) was developed with the purpose of measuring such constructs in students from the 4th to the 9th year of elementary school.

Therefore, the present study aims to verify the psychometric properties of a test constructed to assess reading and executive functions. Our hypothesis is that the AREF test will demonstrate evidence of convergent validity through correlations with already validated tests of reading comprehension and executive functions. Specifically, each AREF subtest (Graphophonological-Semantic Flexibility, Inhibitory Control, Flexibility, and Working Memory) is expected to show correlation with the executive function scores it aims to measure. Given that it is a reading test, we hypothesize that subtest results will exhibit stronger correlations with Verbal IQ than with Performance IQ measures. Additionally, we also hypothesize that the subtests will show good evidence of reliability. Finally, we believe that there will be significant differences in AREF battery performance among different age groups, with superior performances observed in older groups compared to younger ones.

## 2 Methods

### 2.1 Sample

The research involved a sample of 93 participants, all Brazilian nationals, Brazilian Portuguese speakers, enrolled from the 4th to the 9th grade of Elementary School. Both public and private school students took part in the research; however, only students from public schools comprised the sample of 4th and 5th graders. The age range of the participants varied from 8 to 14 years, and all of them were selected from two Brazilian states, Espírito Santo (21.5%) and Minas Gerais (78.5%).

Participants were recruited after the researchers contacted the schools. The institutions that showed interest in participating in the research distributed the Consent Terms to be signed by the students’ parents. School representatives were instructed not to hand out the terms to students who met at least 1 of the following exclusion criteria: (1) manifesting complaints or indicators of visual, auditory, neurological, behavioral, and/or cognitive impairment; (2) receiving a diagnosis of developmental, language, and/or learning disorders; (3) not being duly enrolled in elementary school; (4) absence, objection, or non-participation in all assessment sessions; (5) reporting difficulties in reading; (6) being in a grade not corresponding to chronological age; and (7) not having the consent form signed by the legal guardian.

### 2.2 Procedures

The Ethics Committee in Research of the Universidade Federal de Minas Gerais approved the present study. Upon ethical approval, contact was established with elementary schools, both public and private, to obtain the necessary institutional authorization to conduct the research. In accordance with the guidelines established in Resolution No. 196/96 and Resolution No. 466/2012 of the National Health Council of the Ministry of Health, all invited institutions were required to sign an Institutional Assent Form. Once this authorization was obtained, the school staff were informed in advance about the study objectives and the procedures for selecting participants, aiming to gain their support in students’ adherence to the research. For the subjects’ participation in the study, parents or legal guardians were requested to sign the Informed Consent Form.

The administration of the AREF battery, along with the complementary tests used for validation, was conducted by a team consisting of three psychologists and eight psychology students, all experienced in administering psychometric tests. A criterion was that the researchers responsible for administering the AREF battery were not the same ones who conducted the complementary tests with the same student, ensuring the independence of the assessments.

The administration sessions were scheduled in advance with the schools to ensure that the battery was administered individually to each student. Before starting the test administration, researchers made sure to create a comfortable and age-appropriate environment for the child, with a table and appropriate testing materials.

The administration of the AREF Battery (composed of the Graphophonological-Semantic Flexibility, Inhibitory Control, Flexibility, and Working Memory subtests) and other tests was divided into two sessions, aiming not to remove the student from the classroom for a single prolonged period. These sessions were spread over two consecutive days, with the entire AREF Battery administered on 1 day and the other tests on the other day (not always in that order). The average duration of each administration session ranged from 30 to 40 min, depending on individual performance and specific needs of each student. It is relevant to highlight that the majority of participants showed interest and engagement in the proposed activities, not expressing fatigue during the assessment process.

After the data collection was completed, individual reports were prepared for each student, detailing their performance on the tasks already commercially available (FDT, WASI, WISC-IV Digits, and PROLEC/PROLEC-SE-R), as a counterpart to the participating institutions. These reports were delivered to the schools with the objective not only to provide access to information about the students’ performance but also to understand their individual needs, enabling the planning of personalized educational interventions if necessary.

### 2.3 Assessment of reading and executive functions

The AREF test consists of 4 subtests, each of them assesses specific aspects of reading comprehension and executive functions: the Graphophonological-Semantic Flexibility task, the Inhibitory Control task, the Flexibility task and the Working Memory task. All of them will be described in the next sessions.

#### 2.3.1 Graphophonological-semantic flexibility

The graphophonological-semantic flexibility plays a crucial role in the ability to comprehend words as it allows for the flexibility of semantic and phonological aspects in word reading. This ability contributes to fluent word reading in early readers (Cartwright et al., 2019). The present study investigated the capacity to switch between graphophonological and semantic components of printed words through an adapted task from previous works (Cartwright, 2007; Cartwright et al., 2010). The resources used in this activity consisted of four sets of cards, including a training set and three test sets. Each set contained 12 cards, each with a printed word, allowing classification along two simultaneous dimensions in a 2×2 matrix, considering both the initial phoneme and the word’s meaning. In the exemple set, 12 cards were presented to the student with the instruction:

*“I have here some cards for you to organize. You can sort them in two ways simultaneously: by their initial letter and by their meaning.”* The administrator would take the first card, show the word to the participant, and continue:

“*See, I will place the word MOOSE, which is an animal, up here.*” The word was placed in the upper left quadrant. A new card was taken out and its word was shown. “*The word CAMEL is an animal too; so, I will also place it at the top, like MOOSE, but I cannot place it on the left side, because this side is for words with M, so I will place it here on the right. Note that I cannot place words of the same meaning, representing the same category like ANIMALS, diagonally.*” The administrator would take the next card, show the word to the participant, and continue the instruction: *“The next word is CHERRY. Since it starts with C, I will place it on the right side, like CAMEL, but I cannot place it on the top because I only put animals there, so I will place it here at the bottom. Note that I also cannot place words with the same letter diagonally.”* A new word was shown to the participant [MANGO], and the administrator would ask: “*Where do I place this next word?*” It was expected that the participant would indicate, either physically or verbally, the bottom left corner, corresponding to the row where fruits are and the column where words with M are. If they gave the correct answer, the administrator would congratulate them and ask why they chose that space.

**Figure 1:**
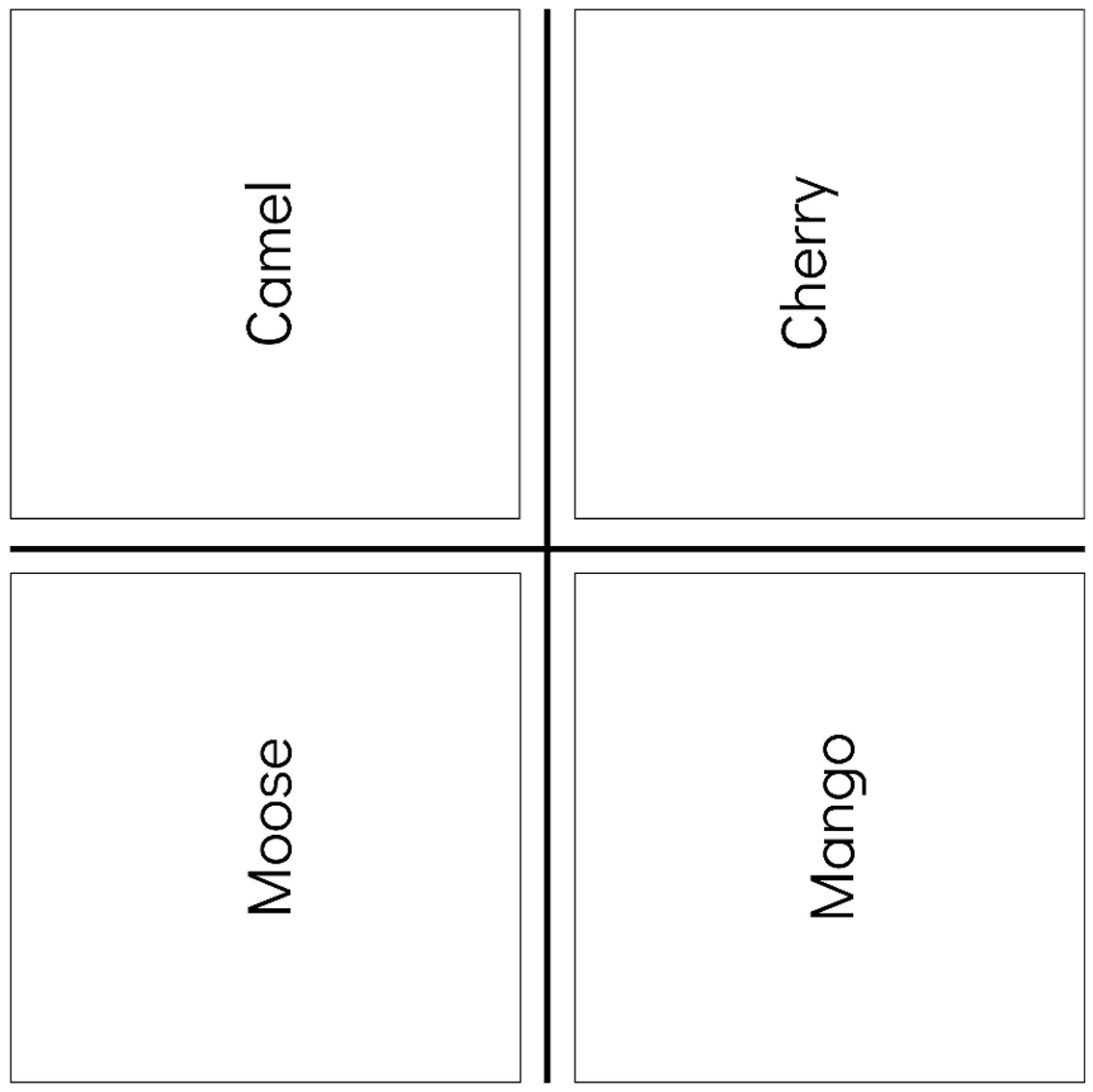
Example of classifying 4 words in the Graphophonological-Semantic Flexibility subtest matrix, simultaneously considering the meaning of the words and the initial letters.

In the justification, it was expected that the student’s response would encompass the division of words in the matrix, simultaneously considering the initial letter and the meaning. For example: “At the top I put the animals and at the bottom the fruits. On the left side, I placed the words with M and on the right side the words with C”. Figure 1 shows an example of a possible classification expected in this task, in which on the left side of the matrix there are only words starting with the letter M, on the right side there are only words starting with the letter C, on the top there are only words animals, and in the lower part only the fruits.

After the administrator classified the first 3 words, explaining the rule, and after the participant classified the fourth word, the administrator would hand over the other 8 cards from this set for the student to perform the classifications. When the student performed the task correctly, they were congratulated, and when the execution was incorrect, the administrator would say “*Not quite*” and reinforce the classification rule.

After the training set, the first test set was conducted, preceded by the instruction: “*Very well. Now I will give you other words, and you will separate them the same way we did until now: by the letter and the meaning. The letters and meanings will not always be the same as the ones we did until now, but you can separate them the same way. If you make a mistake and want to change a word, continue the activity, and you can change the word’s place at the end. You may begin.*” After classifying this set, the administrator would ask why the participant organized the words that way. Again, it was expected that they would respond that they considered, simultaneously, the initial letters and the meaning of the words.

Results were recorded in terms of the time required to classify each set of words, along with the assembly of the matrix (1 point when correct and 0 when incorrect), followed by justifications for their classifications (2 points when correct and 0 when incorrect). Once the test items were scored, the administrator did not provide feedback on the participant’s performance.

To ensure the standardization of the test application, each set of cards was always presented in the same sequence, specifically test sets 1, 2, and 3. Additionally, the sequence of words within each set remained constant throughout the study.

Scoring followed the following criteria: one point was awarded for the accurate assembly of the matrix, and two points were assigned for an adequate justification of the process performed. Considering that there were 3 items scored, the maximum score obtained in this subtest was 9. In cases where there was an error in both stages of the activity, the score was null.

The study took into account the characteristics of the language used in the stimuli, such as high-frequency orthographic words in Brazilian Portuguese. This selection was conducted using data repositories available on the platforms http://lexicodoportugues.com/ and https://www.corpusdoportugues.org/now/. The selection criteria included syllabic length (disyllabic, trisyllabic, and polysyllabic) and the complexity of words according to the structure of the initial syllable. Polysemous, homographic, and monosyllabic words were deliberately excluded. The mentioned guidelines ensured the diversity of the chosen words by varying in regularity, length, and syllabic complexity.

#### 2.3.2 Inhibitory control

The inhibitory control subtest assesses the student’s ability to suppress automatic responses and resist distractions during reading. Divided into three stages (1 baseline and 2 inhibition), the participant is instructed to read narrative texts aloud, all consisting of 94 words each. After each reading, the participant must retell the events in the story and orally answer three specific questions about the text. The answers are definitive and require the direct retrieval of the information read, without the need for inferences from secondary information. The retelling involves identifying eight specific events, which are recorded in the response booklet as a checklist. Each event remembered in the retelling is counted as 1 point, as well as each correct answer. The maximum possible score for each stage of the subtest is 11 points, and the reading time for each text is timed.

**Figure 2:**
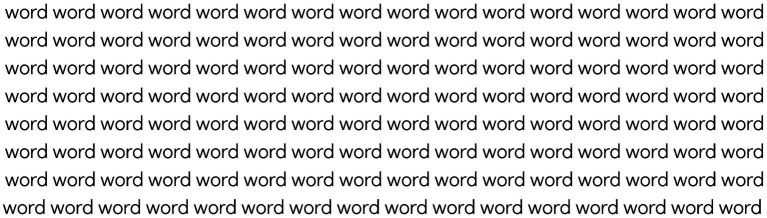
Example of text without interference from other colors used as a baseline for the AREF Inhibitory Control and Flexibility subtests.

The first stage consists of a typical text without interference from other colors, as illustrated in Figure 2, designed to assess reading fluency and text comprehension, measured, respectively, by the reading time and the score obtained from the retelling and responses to the questions. The results of this stage are used as a baseline for comparison with the results of inhibitory control and flexibility. Therefore, this task is called Baseline Text (BT).

**Figure 3:**
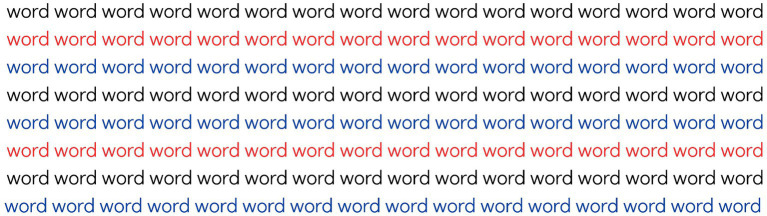
Example of text from the first Inhibitory Control task of the AREF test, in which the participant must read only the black lines.

The second stage begins with a preliminary training task, where the participant is presented with a text containing lines in three different colors: blue, red, and black, as shown in Figure 3. The participant is instructed to read aloud only the black sentences. After reading this training text, the evaluation text is displayed, and the instructions are reiterated. The reading time is timed in seconds from the start of reading and is stopped upon completion. The time is recorded in the response booklet, as well as the number of incorrectly read words, the number of colored sentences read, and the number of black lines ignored. After this stage, the participant is again invited to retell the story and orally answer three questions about the text read, and the score is recorded in the booklet based on the participant’s performance. This is the first task that seeks to evaluate Inhibitory Control (IC-1).

**Figure 4:**
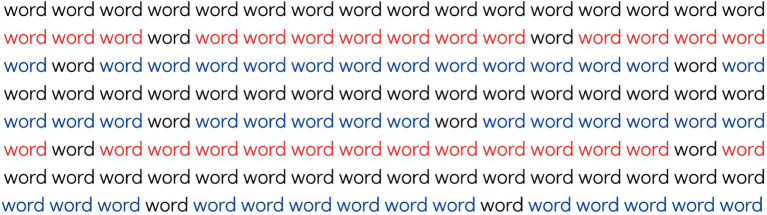
Example of text from the second Inhibitory Control task of the AREF test, in which the participant must read only the black lines while avoiding reading words written in black on colored lines.

In the third stage, a text with lines of different colors is presented once again, serving as further training before the official task. The examiner instructs the participant to continue reading only the sentences in black, avoiding reading sentences in other colors. Additionally, there are black words within colored sentences, which should not be read, as shown in Figure 4. The participant must only read aloud the sentences in which all words are black. Similar to the previous stages, the reading time is timed in seconds from the beginning and stopped upon completion. The time is recorded in the response booklet. Accuracy is also recorded, noting any instances where the participant read words that should have been ignored, including errors of reading black words within colored sentences and any failures involving reading lines in colors other than black. After reading, the participant is asked to retell the story and orally answer three questions about the text read. Both correct and incorrect responses are recorded in the response booklet. This is the second task that seeks to evaluate Inhibitory Control (IC-2).

#### 2.3.3 Flexibility

**Figure 5:**
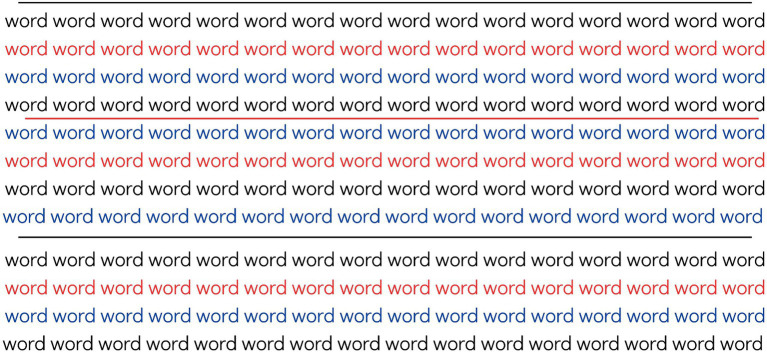
Example of text from the AREF Flexibility task, in which the participant must alternate reading between sentences of different colors depending on the color of the visual sign (line) present in the text.

In the Flexibility (FL) subtest, the participant is required to alternate reading sentences of different colors according to a visual cue, aiming to assess the schoolchild’s cognitive flexibility. At the beginning of this task, before the sentences that comprise the text, there is a continuous black line, as shown in Figure 5. This black line indicates that the participant should read only the black sentences, ignoring the red or blue sentences. The reading of the black sentences should continue until the appearance of another visual cue indicating a change in the color of the sentences to be read. In Figure 5, as in the original task, this cue is represented by a red line, after which the participant should read only the red sentences. The reading of the red sentences should continue until a new visual cue indicates a change in color. In Figure 5, as in the original task, this cue is the second black line. The test is preceded by a training item. The reading time for this subtest is recorded, followed by the retelling of the story and responses to three specific questions. Responses are scored based on the direct retrieval of the information read, with a total of 8 events to be identified during the retelling, similar to the baseline text and the inhibitory control tasks.

#### 2.3.4 Working memory

In the Working Memory subtest, the examiner instructs the participant to read aloud sentences and, after reading, to retell the story in reverse order, without the visual aid of the text. Initially, a practice session with a text consisting of 2 events is conducted, followed by the commencement of the evaluative task. An example of a stimulus text for practice and the expected response after reading is as follows: The stimulus text ‘I went to the park. I played soccer.’ is provided, and the expected response is ‘I played soccer. Prior to that, I went to the park’.

In total, after the practice session, participants were provided with seven different texts to read aloud. These narrative texts, which contained words commonly used in Brazilian Portuguese, varied in content and in the number of events included. The first text presented three events, and each subsequent text introduced one additional event compared to its predecessor, thereby gradually increasing the demand for information retrieval (span). Each sentence in the texts, ending with a period, represents an event to be remembered. Each event remembered correctly corresponds to one point. However, once an event is remembered and reported, any other event will only be scored if it chronologically preceded it.

In composing the original texts in the Working Memory subtest, the following criteria were applied: segmentation of events through periods and ensuring consistency in the length of sentences.

### 2.4 Neuropsychological protocol

To establish the construct validity of the instrument developed to concurrently assess reading and executive functions, analyses were conducted to verify evidence of external construct validity. For this purpose, the following instruments were used: the Vocabulary and Matrix Reasoning subtests of the WASI (Wechsler, 2014); the PROLEC Text Comprehension (Capellini et al., 2012) for 4th and 5th-grade students; the PROLEC-SE-R Narrative Comprehension for 6th to 9th-grade students (Cuetos et al., 2022); the Five Digit Test (FDT) (Sedó et al., 2015); and the Digit Span subtest of the WISC-IV (Wechsler, 2013).

#### 2.4.1 Wechsler abbreviated scale of intelligence

To assess general intelligence across a wide age range, the Wechsler Abbreviated Scale of Intelligence (WASI) was utilized, comprising the Vocabulary and Matrix Reasoning subtests. Whereas the Vocabulary subtest assesses verbal comprehension and knowledge of word meanings, Matrix Reasoning evaluates nonverbal fluid reasoning through visual patterns.

Individuals who scored below 70 on the IQ test were excluded, leading to the elimination of one participant.

#### 2.4.2 PROLEC’s text comprehension

The PROLEC assesses reading processes in children from 2nd to 5th grade of elementary school. The subtest consists of four brief texts, followed by questions addressing both literal and inferential aspects of textual comprehension. Each text has 4 questions, totaling 16 questions distributed among the texts. A score of 1 point is assigned to each correct answer, while incorrect answers receive 0 points, allowing participants to obtain a maximum of 16 points.

#### 2.4.3 PROLEC-SE-R’s narrative comprehension

To assess narrative reading comprehension in later grades, the PROLEC-SE-R was employed, targeting students from 6th to 9th grade of elementary school and from 1st to 3rd grade of high school. This instrument involves reading a narrative text followed by 10 multiple-choice questions, with the allowance to consult the text during the assessment.

#### 2.4.4 Five digit test

Another instrument employed was the Five Digits Test. This test assesses inhibitory control and cognitive flexibility. It comprises four distinct stages. In the first stage, named Reading, participants are presented with rectangles containing numerals from 1 to 5, with the quantity of numerals inside the rectangle corresponding to the magnitude of the represented number (e.g., two numerals inside the rectangle for the number 2). The objective is for the participant to name the numerals contained in 50 stimuli as quickly as possible. In the second stage, Counting, the rectangles contain up to five asterisks, and participants must count the quantity of asterisks in 50 stimuli as quickly as possible. The third stage, called Choosing, repeats the presentation of the rectangles, but this time with an incongruent condition, meaning the quantity of numerals inside the rectangle does not match the magnitude of the number (e.g., three numerals 4 inside the rectangle). Participants must count the quantity of numerals in 50 stimuli as quickly as possible, inhibiting the automatic response of pronouncing the name of the represented numeral. In the fourth stage, Shifting, participants continue counting the quantity of numerals, but when presented with a rectangle with a thicker border, they must say the name of the numeral contained. Thus, counting and naming responses are alternated. Also, 50 stimuli are presented in this stage. In addition to the four mentioned stages, the test provides measures of inhibition and flexibility, derived from the time spent in Stages 1, 3, and 4. The inhibition measure is calculated by subtracting the time from Stage 1 (Reading) from the time from Stage 3 (Choosing). The flexibility measure is calculated by subtracting the time from Stage 1 (Reading) from the time from Stage 4 (Shifting). Test correction considers the total time taken for each stage, as well as the quantity of errors made.

#### 2.4.5 Digit span subtest (WISC-IV)

The “Digit Span” subtest of the WISC-IV was employed to assess working memory and auditory attention in children. In this subtest, the examiner presents a series of digits for the participant to repeat either in the same order (Forward) or in reverse order (Backward), with a gradual increase in difficulty.

### 2.5 Data analysis

All analyses were carried out considering the total results of each subtest of AREF. For the GSF, IC, and FL tasks, in which the duration of execution was measured, the final score of each subtest was calculated considering both accuracy (number of correct responses) and time taken. This approach is supported by evidence in the literature indicating that, in both executive function and reading tests, time is a crucial variable for predicting performance. For example, Magnus et al. (2019) demonstrated that the joint use of accuracy and reaction time improves the precision of inhibitory control measurement compared to models that use only accuracy. Similarly, Su and Davison (2019) noted that including response time measures can improve the validity of a reading test, as lower response times during reading are observed in individuals with higher ability. Therefore, we opted to include both accuracy and time in our scoring approach to ensure a more accurate and valid assessment of performance.

Performance on the GSF task was evaluated by summing the Execution Points (EP) and Justification Points (JP) of the three items composing the task, multiplied by 60, and divided by the sum of the time (T) of the three items. This evaluation resulted in the efficiency score (GSF-ES) in task execution, as demonstrated by the formula below:


$$\begin{array}{l} GSF\;\text{Efficiency}\;\text{Score}=\\ \left(EP1+JP1+EP2+JP2+EP3\;+JP3\;\right)\;x\;60/\left(T1\;+T2\;+T3\;\right) \end{array}$$

Regarding AREF’s Inhibitory Control and Flexibility subtests, statistical analyses were also conducted considering the efficiency score obtained in each activity, using the following calculation: (Recall points + response points) x 60 / reading time in seconds. As a result, there were four efficiency scores in that stage: from Baseline Text (BT-ES), from the first Inhibitory Control task (IC1-ES), from the second Inhibitory Control task (IC2-ES), and from Flexibility task (FL-ES).

The result of the Working Memory task (WM Total) was calculated by summing the results of the seven items composing the task:


WMTotal=WM1+WM2+WM3+WM4+WM5+WM6+WM7

To verify evidence of convergent validity, it was examined the relationship between AREF subtests results and external measures with correlation analysis. Prior to this analysis, the multivariate Shapiro–Wilk test was applied, indicating that the joint distributions of the variables were non-parametric, justifying the use of Spearman correlation.

For correlation analysis, participants’ results on external measures were transformed into scores or ratings obtained from the respective tasks. Regarding WASI, T-scores of the applied subtests (Vocabulary and Matrix Reasoning) were utilized. Regarding the FDT, inhibition and flexibility percentiles were used. As for the WISC-IV Digit Span subtest, both forward and backward span, as well as Scaled Scores (SS) obtained throughout the task, were employed. Concerning the reading comprehension subtests of the PROLEC and PROLEC-SE-R tests, their classifications based on individual performance had to be unified. In PROLEC, administered in the 4th and 5th grades, the categories are “SD” (Severe Difficulty), “D” (Mild Difficulty), and “A” (Average), whereas in PROLEC-SE-R, administered from the 6th to 9th grades, the categories include “SD” (Severe Difficulty), “D” (Mild Difficulty), “L” (Low), “A” (Average), and “H” (High). As our analyses involved the entire population from the 4th to 9th grades, the “Low,” “Average,” and “High” categories from PROLEC-SE-R were grouped into a single category, corresponding to the “Average” classification of PROLEC. This approach was adopted to standardize categories and ensure greater precision in statistical analyses involving both population groups.

Given that AREF subtests measure distinct constructs, some observations are needed. Firstly, the correlations conducted for the GSF efficiency score were the same for Inhibitory Control and Flexibility tasks, which included reading comprehension measures (PROLEC and PROLEC-SE-R subtests), executive function measures (Flexibility and Inhibition percentiles of the FDT), and verbal and performance measures of the WASI (Vocabulary subtest and Matrix Reasoning subtest, respectively). Secondly, correlations of WM Total were performed with the same aforementioned reading comprehension measures, Working Memory measures (forward and backward span, in addition to the Scaled Scores), and verbal and performance measures of the WASI as well.

In this study, it was also investigated the differences between the mean performance on the Baseline Text task and the other tasks of the Inhibitory Control and Flexibility subtests. Before comparison, the Shapiro–Wilk test was applied to check the data distribution. In cases where the distribution was non-parametric, the Wilcoxon test was used for comparison, while the effect size was evaluated by the Point-Biserial Correlation Coefficient. When the distribution was parametric, the paired t-test was employed, with the effect size calculated by Cohen’s d test. The comparisons made were between the group’s efficiency performance in the Baseline Text and efficiency in IC-1, IC-2, and FL. These comparisons were feasible because all tests shared the same efficiency calculation and the same format, including the same number of words in the target texts and the same amount of clauses to be retold and questions to be answered.

To strengthen the evidence of construct validity, an investigation was conducted on potential differences in the performances of distinct groups. The instrument’s ability to differentiate these groups provides evidence of concurrent validity, which is used to evaluate test-criterion relationships (American Educational Research Association, 2014), where the scores obtained on the tasks predict outcomes observed at the time of test administration.

Considering that executive function and reading comprehension skills improve throughout schooling, differences in the performance of individuals from different school years on the AREF subtests were measured. To evaluate the performance of groups from different school years on the AREF subtests, Analysis of Variance (ANOVA) was applied. Detailed group comparison analysis was conducted only when the ANOVA indicated statistical significance (*p* < 0.05), meaning significant differences were detected between the groups. In such cases, the Levene’s test was employed to examine the homogeneity of variances among the groups. If Levene’s test revealed a *p* > 0.05, a *Post Hoc* analysis using Tukey’s test was performed to identify which groups showed significant differences in performance.

Given that previous studies have identified significantly different performances between students from public and private schools in reading comprehension (Marques de Oliveira et al., 2024; Cáceres-Serrano and Alvarado-Izquierdo, 2017; Çigdemir and Akyol, 2022) and executive functions (Jacobsen et al., 2017), the AREF scores of participants from both school types were compared. To perform this comparison, the normality of the data distribution was first assessed using the Shapiro–Wilk test. If the distribution was non-parametric, the Mann–Whitney test was employed. When parametric distribution was confirmed, Levene’s test was used to evaluate the equality of variances. In cases where Levene’s test did not show significance, the independent samples t-test was subsequently applied, with effect size estimated using Cohen’s *d*. As previously mentioned, the sample of 4th and 5th-grade students consisted exclusively of public school students. To eliminate the possibility that differences in performance between public and private schools were due to the younger average age of public school students, the comparison between school types was conducted only for students from 6th to 9th grade (N = 50). The identification of performance differences between students from public and private schools on the AREF test also contributes as evidence of concurrent validity.

The internal consistency of each AREF subtest was assessed using Cronbach’s alpha coefficient. It should be noted that, once Inhibitory Control subtest and Flexibility subtest were made up of a similar structure (recall points, response points and reading time), and, besides that, required almost the same cognitive constructs, these subtests were grouped in this internal consistency analysis.

All statistical analyses were performed using JASP 0.17.2.0 software (JASP Team, 2023).

## 3 Results

The characteristics of the participants are presented in Table 1, which includes information on age, gender, grade level, and the type of school within the collected sample.

**Table 1:** Characterization of participant profiles (*n* = 93) according to age range, gender, grade level and type of school.

Feature	Category	No.	%
Age	8 years	1	1,1%
	9 years	17	18,3%
	10 years	26	28%
	11 years	12	12,9%
	12 years	14	15,1%
	13 years	14	15,1%
	14 years	9	9,7%
Gender	Male	37	39,8%
	Female	56	60,2%
Grade level	4th grade	17	18,3%
	5th grade	26	28,0%
	6th grade	15	16,1%
	7th grade	16	17,2%
	8th grade	13	14,0%
	9th grade	6	6,5%
Type of school	Public	61	65,6%
	Private	32	34,4%

### 3.1 Construct validity

Table 2 illustrates the Spearman correlation of efficiency scores obtained in the Graphophonological-Semantic Flexibility subtest with the classification of results from the PROLEC and PROLEC-SE-R subtests, along with the percentiles of inhibition and flexibility from the FDT, and the T-scores of the Vocabulary and Matrix Reasoning subtests of the WASI.

**Table 2:** Spearman correlation of the efficiency of the graphophonological-semantic flexibility subtest of AREF with the classification of PROLEC and PROLEC-SE-R and with the percentiles of inhibition and flexibility of FDT.

Variable		GSF-ES
PROLEC classification	Spearman	0.355
	*p*-value	<0.001***
Inhibition - PC	Spearman	0.209
	*p*-value	0.045*
Flexibility - PC	Spearman	0.116
	*p*-value	0.266
WASI vocabulary	Spearman	0.348
	*p*-value	<0.001***
WASI matrix reasoning	Spearman	0.109
	*p*-value	0.298

*p < 0.05; **p < 0.01; ***p < 0.001. PROLEC Classification, Classification of PROLEC and PROLEC-SE-R. Inhibition – PC, Percentile data of inhibition from FDT. Flexibility – PC, Percentile data of flexibility from FDT. GSF-ES, Efficiency score of the graphophonological-semantic flexibility subtest.

The results indicate that the efficiency score obtained in the GSF task presented weak, but significant, positive correlations with the FDT Inhibition percentile [rs (93) = 0.209; *p* = 0.045]. Likewise, the correlations of the GSF subtest efficiency scores were positive and significant with the classification obtained in the PROLEC and PROLEC-SE-R tests, of moderate magnitude [rs (91) = 0.355; *p* < 0.001], as well as with the T-score of the WASI Vocabulary subtest [rs (91) = 0.348; *p* < 0.001]. No significant correlations were found between the GSF subtest and the FDT Flexibility percentile [rs (93) = 0.116; *p* = 0.266] and the WASI Matrix Reasoning T-score [rs (93) = 0.109; *p* = 0.298].

Table 3 depicts the Spearman correlation of scores from the Inhibitory Control and Flexibility subtests of the AREF with the classifications of results from the PROLEC and PROLEC-SE-R subtests, along with the percentiles of inhibition and flexibility from the FDT, and the T-scores of the Vocabulary and Matrix Reasoning subtests of the WASI.

**Table 3:** Spearman correlation of the efficiency of tasks from the baseline text, the inhibitory control and the flexibility subtests of AREF with the T score of the vocabulary and matrix reasoning subtests of WASI and the percentile of inhibition and flexibility from FDT.

Variable		BT-ES	IC1-ES	IC2-ES	FL-ES
PROLEC classification	Spearman	0.339	0.367	0.339	0.358
	*p*-value	<0.001***	<0.001***	<0.001***	<0.001***
Inhibition - PC	Spearman	0.300	0.387	0.284	0.358
	*p*-value	0.004**	<0.001***	0.006**	<0.001***
Flexibility - PC	Spearman	0.112	0.265	0.203	0.100
	*p*-value	0.286	0.010*	0.051	0.341
WASI vocabulary	Spearman	0.307	0.412	0.390	0.262
	*p*-value	0.003**	<0.001***	<0.001***	0.011*
WASI matrix reasoning	Spearman	0.131	0.172	0.124	0.185
	*p*-value	0.211	0.099	0.236	0.076

*p < 0.05; **p < 0.01; ***p < 0.001. PROLEC Classification, Classification of PROLEC and PROLEC-SE-R. Inhibition – PC, Percentile data of inhibition from FDT. Flexibility – PC, Percentile data of flexibility from FDT. TB-ES, Baseline text efficiency. IC1-ES, Efficiency of Text 1 from inhibitory control subtest. IC2-ES, Efficiency of Text 2 from inhibitory control subtest. FL-ES, Efficiency of the flexibility subtest.

As expected, the efficiency scores in all AREF texts showed a positive and significant correlation, of moderate magnitude, with the classification of performance in the PROLEC and PROLEC-SE-R subtests (ranging from 0.339 to 0.367). The AREF scores also showed positive and significant correlations, of weak to moderate magnitude, with the FDT Inhibition percentile (ranging from 0.284 to 0.387), as well as with the WASI Vocabulary subtest T-score (ranging from 0.262 to 0.412). Only the first inhibitory control task of the AREF test showed a positive and significant correlation, of weak magnitude, with the FDT flexibility percentile [rs (93) = 0.265; *p* < 0.010]. There was no significant correlation between the AREF subtests and the T-score of the Matrix Reasoning subtest of the WASI.

The results showed that the total score of the AREF working memory task correlated significantly and positively with the classification of the PROLEC and PROLEC-SE-R subtests [moderate magnitude, rs (93) = 0.365; *p* < 0.001], with the Scaled Scores of the WISC-IV Digit Span subtest (weak magnitude, rs (93) = 0.259; *p* = 0.012), with the direct span of the WISC-IV Digit Span subtest (moderate magnitude, rs (93) = 0.396; *p* < 0.001), and with the T-score of the Vocabulary subtests (moderate magnitude, rs (93) = 0.328; *p* < 0.001) and Matrix Reasoning [weak magnitude, rs (93) = 0.241; *p* = 0.020] from WASI. These data are presented in Table 4.

**Table 4:** Spearman correlation of the score obtained in the working memory subtest of AREF with the result classifications of PROLEC and PROLEC-SE-R subtests, with the digit span scaled scores, of the WISC-IV, and with the T-score of the vocabulary and matrix reasoning subtests of the WASI.

Variable		WM total
PROLEC classification	Spearman	0.365
	*p*-value	<0.001***
Digit span - SS	Spearman	0.259
	*p*-value	0.012*
Forward span	Spearman	0.396
	*p*-value	<0.001***
Backward span	Spearman	0.160
	*p*-value	0.125
WASI vocabulary	Spearman	0.328
	*p*-value	0.001**
WASI matrix reasoning	Spearman	0.241
	*p*-value	0.020*

*p < 0.05; **p < 0.01; ***p < 0.001. WM Total, total points obtained in the 7 items of the working memory task of AREF. Digits – SS, Scaled scores data of digit span from WISC-IV.

The comparison of efficiency between the Baseline Text and IC-1 Text was conducted using the Paired Wilcoxon Test, due to the non-parametric distribution. For other comparisons with parametric distribution, independent samples t-tests were employed. No significant difference was observed when comparing the performance in the Baseline Text to the IC-1 Text (U = 2015.000; *p* = 0.898) or to the FL Text [*t* (91) = 1.849; *p* = 0.068]. However, a significant difference was identified [*t* (91) = 2.098; *p* = 0.039] between the performance in the Baseline Text (M = 10.2, SD = 5.5) and the performance in the IC-2 Text (M = 9.2, SD = 5.8), with a small effect size (d = 0.218).

Regarding the performance analyses of different school years, the ANOVA results revealed a significant group effect on all subtests. Effects were observed in the GSF subtest, *F* (5, 89) = 8.115, *p* < 0.001, η^2^ = 0.318, as well as in IC-1, *F* (5, 89) = 10.898, *p* < 0.001, η^2^ = 0.385, and in IC-2, *F* (5, 89) = 195.484, *p* < 0.001, η^2^ = 0.312. Significant differences were also observed in group performance in the Flexibility subtest, *F* (5, 89) = 120.331, *p* < 0.001, η^2^ = 0.242, and in the WM subtest, *F* (5, 89) = 10.345, *p* < 0.001, η^2^ = 0.373. In all tasks, significant group differences occurred in most comparisons between students from the 4th and 5th grades and those from other school years. The results of the Analysis of Variance are presented in Table 5, and the comparisons of the different school years in each of the subtests are shown in Tables 6–10.

**Table 5:** Results of the analysis of variance (ANOVA) for comparison of different school grades in relation to AREF subtests.

Cases	Sum of scores	df	Mean of scores	*f*	*p*	η^2^
Grade - GSF efficiency	65.670	5	13.134	8.115	<0.001***	0.318
Residuals	140.801	87	1.618			
Grade - IC-1 efficiency	777.688	5	155.538	10.898	<0.01**	0.385
Residuals	1241.629	87	14.272			
Grade - IC-2 efficiency	977.421	5	195.484	7.888	<0.001***	0.312
Residuals	2155.993	87	24.782			
Grade - FL efficiency	601.656	5	120.331	5.540	<0.001***	0.242
Residuals	1889.571	87	21.719			
Grade - total WM	2505.078	5	501.016	10.345	<0.001***	0.373
Residuals	4213.653	87	48.433			

*p < 0.05; **p < 0.01; ***p < 0.001. F represents the F statistic of the ANOVA, and p denotes the significance value associated with the analysis. GSF Efficiency, Efficiency of the graphophonological-semantic flexibility subtest. IC-1 Efficiency, Efficiency of the first text of the inhibitory control subtest. IC-2 Efficiency, Efficiency of the second text of the inhibitory control subtest. FL Efficiency, Efficiency of the flexibility subtest. Total WM, Total points obtained in the 7 items of the AREF working memory task.

**Table 6:** Results of *post hoc* comparisons of analysis of variance (ANOVA) comparing the performance of different school years (4th to 9th grade) on the GSF subtest of AREF.

Grade		Mean difference	SE	*t*	p_tukey_
4	5	0.084	0.397	0.211	1.000
	6	−1.553	0.451	−3.447	0.011
	7	−1.285	0.443	−2.900	0.052
	8	−1.918	0.469	−4.092	0.001
	9	−1.804	0.604	−2.986	0.041
5	6	−1.637	0.412	−3.970	0.002
	7	−1.369	0.404	−3.387	0.013
	8	−2.002	0.432	−4.633	<0.001
	9	−1.888	0.576	−3.276	0.018
6	7	0.268	0.457	0.587	0.992
	8	−0.365	0.482	−0.756	0.974
	9	−0.250	0.615	−0.407	0.999
7	8	−0.633	0.475	−1.333	0.766
	9	−0.519	0.609	−0.852	0.957
8	9	0.114	0.628	0.182	1.000

*P-value adjusted for comparing a family of 6.*

**Table 7:** Results of *post hoc* comparisons of analysis of variance (ANOVA) comparing the performance of different school grades (4th to 9th) on the IC-1 subtest of AREF.

Grade		Mean difference	SE	*t*	p_tukey_
4	5	−0.185	1.178	−0.157	1.000
	6	−5.396	1.338	−4.032	0.002
	7	−5.338	1.316	−4.057	0.001
	8	−6.133	1.392	−4.406	<0.001
	9	−7.480	1.794	−4.170	<0.001
5	6	−5.212	1.225	−4.255	<0.001
	7	−5.153	1.200	−4.293	<0.001
	8	−5.948	1.283	−4.635	<0.001
	9	−7.295	1.711	−4.264	<0.001
6	7	0.058	1.358	0.043	1.000
	8	−0.737	1.432	−0.515	0.995
	9	−2.084	1.825	−1.142	0.862
7	8	−0.795	1.411	−0.564	0.993
	9	−2.142	1.808	−1.184	0.843
8	9	−1.347	1.865	−0.722	0.979

*P-value adjusted for comparing a family of 6.*

**Table 8:** Results of *post hoc* comparisons from analysis of variance (ANOVA) comparing the performance of different school grades (4th to 9th) on the IC-2 subtest of AREF.

Grade		Mean difference	SE	*t*	p_tukey_
4	5	−1.229	1.553	−0.791	0.968
	6	−5.325	1.763	−3.020	0.038
	7	−7.380	1.734	−4.256	<0.001
	8	−7.964	1.834	−4.342	<0.001
	9	−7.941	2.364	−3.359	0.014
5	6	−4.096	1.614	−2.538	0.125
	7	−6.151	1.582	−3.889	0.003
	8	−6.736	1.691	−3.983	0.002
	9	−6.713	2.255	−2.977	0.042
6	7	−2.055	1.789	−1.149	0.859
	8	−2.640	1.886	−1.399	0.727
	9	−2.616	2.405	−1.088	0.885
7	8	−0.585	1.859	−0.315	1.000
	9	−0.561	2.383	−0.236	1.000
8	9	0.023	2.457	0.009	1.000

*P-value adjusted for comparing a family of 6.*

**Table 9:** Results of *post hoc* comparisons of analysis of variance (ANOVA) comparing the performance of different school grades (4th to 9th) on the FL subtest of AREF.

Grade		Mean difference	SE	*t*	p_tukey_
4	5	−0.058	1.454	−0.040	1.000
	6	−3.532	1.651	−2.139	0.277
	7	−5.172	1.623	−3.186	0.024
	8	−5.404	1.717	−3.147	0.027
	9	−6.639	2.213	−3.000	0.040
5	6	−3.475	1.511	−2.299	0.206
	7	−5.115	1.481	−3.454	0.011
	8	−5.347	1.583	−3.377	0.014
	9	−6.582	2.111	−3.118	0.029
6	7	−1.640	1.675	−0.979	0.923
	8	−1.872	1.766	−1.060	0.896
	9	−3.107	2.251	−1.380	0.739
7	8	−0.232	1.740	−0.133	1.000
	9	−1.467	2.231	−0.657	0.986
8	9	−1.235	2.300	−0.537	0.994

*P-value adjusted for comparing a family of 6.*

**Table 10:** Results of *post hoc* comparisons of analysis of variance (ANOVA) comparing the performance of different school grades (4th to 9th) on the WM subtest of AREF.

Grade		Mean difference	SE	*t*	p_tukey_
4	5	−1.152	2.171	−0.531	0.995
	6	−8.749	2.465	−3.549	0.008
	7	−11.570	2.424	−4.773	<0.001
	8	−11.575	2.564	−4.514	<0.001
	9	−12.716	3.305	−3.848	0.003
5	6	−7.597	2.256	−3.367	0.014
	7	−10.418	2.211	−4.711	<0.001
	8	−10.423	2.364	−4.409	<0.001
	9	−11.564	3.152	−3.669	0.005
6	7	−2.821	2.501	−1.128	0.869
	8	−2.826	2.637	−1.071	0.891
	9	−3.967	3.362	−1.180	0.845
7	8	−0.005	2.599	−0.002	1.000
	9	−1.146	3.332	−0.344	0.999
8	9	−1.141	3.435	−0.332	0.999

*P-value adjusted for comparing a family of 6.*

Regarding the comparison between public and private schools, it was found that the data distributions in GSF, IC-2 and WM were parametric and exhibited equal variances. Therefore, the comparison between the groups was conducted using the Student’s t-test. A significant difference in WM performance was observed between the groups [*t* (48) = −2.135; *p* = 0.038], with a medium effect size indicated by Cohen’s d of −0.629, showing higher performance by students from private schools (Mean = 29.3, Standard Deviation = 5.96) compared to those from public schools (Mean = 25.5, Standard Deviation = 6.51). No significant difference was found in GSF performance [*t* (48) = −0.792; *p* = 0.433] or IC-2 performance [*t* (48) = −1.477; *p* = 0.146]. The detailed results are presented in Table 11, with the magnitude of the means described in Table 12. Supplementary Figure S1, illustrates the comparison of the average performance of individuals from the two groups in the WM task.

**Table 11:** Comparative analysis using student’s *t*-test of performance in GSF, IC-2 and WM subtests between students from private and public schools in grades 6th to 9th.

Subtest	*t*	df	*p*	Cohen’s d	SE Cohen’s d
GSF efficiency	−0.792	48	0.433	−0.233	0.297
IC-2 efficiency	−1.477	48	0.146	−0.435	0.303
Total WM	−2.135	48	0.038*	−0.629	0.313

*p < 0.05; **p < 0.01; ***p < 0.001. GSF Efficiency, Efficiency of graphophonological-semantic flexibility subtest. IC-2 Efficiency, Efficiency of the second task of inhibitory control subtest. Total WM, Total points obtained in the 7 items of the AREF working memory task.

**Table 12:** Means and standard deviations for public and private school participants (6th to 9th grade) on GSF, IC-2, and WM subtests.

Subtest	School type	*N*	Mean	Standard deviation
GSF Efficiency	Public	18	2.748	1.314
	Private	32	3.069	1.414
IC-2 efficiency	Public	18	10.646	4.862
	Private	32	13.043	5.830
Total WM	Public	18	25.500	6.510
	Private	32	29.375	5.961

GSF Efficiency, efficiency of graphophonological-semantic flexibility subtest. IC-2 Efficiency, Efficiency of the second task of inhibitory control subtest. Total WM, Total points obtained in the 7 items of the AREF working memory task.

In contrast to the other subtests, the analysis of efficiency score distributions for IC-1 and FL between public and private schools revealed them to be non-parametric. Therefore, Mann–Whitney tests were applied for the analyses. The results indicated statistically significant differences in both IC-1 task (w = 187.500, *p* = 0.043) and FL task (w = 190.000, *p* = 0.049). Rank-Biserial correlations showed medium effect sizes of −0.349 for IC-1 and -0.340 for FL. Participants from private schools demonstrated higher performance compared to those from public schools in the IC-1 task (Mdn private = 13.605 vs. Mdn public = 11.325), as well as in the FL task (Mdn private = 12.700 vs. Mdn public = 9.575). The analysis results are presented in Table 13, and the medians for each group in each subtest are shown in Table 14. Supplementary Figures S2, S3, illustrate, respectively, the comparison of students’ performance between school types in the IC-1 and FL tasks.

**Table 13:** Comparison between public and private schools with participants from 6th to 9th grade - Mann–Whitney test for IC-1 and FL subtests.

Subtest	*w*	*p*	Rank-Biserial correlation
IC-1 efficiency	187.500	0.043*	−0.349
FL efficiency	190.000	0.049*	−0.340

*p < 0.05; **p < 0.01; ***p < 0.001. IC-1 Efficiency, Efficiency of the first task of inhibitory control subtest. FL Efficiency, Efficiency of the flexibility subtest.

**Table 14:** Medians and standard deviations for public and private school participants (6th to 9th grade) on IC-1 and FL subtests.

Subtest	School type	*N*	Median	Standard deviation
IC-1 Efficiency	Public	18	11.325	4.192
	Private	32	13.605	3.601
FL Efficiency	Public	18	9.575	4.841
	Private	32	12.700	5.381

*p < 0.05; **p < 0.01; ***p < 0.001. IC-1 Efficiency, Efficiency of the first task of inhibitory control subtest. FL Efficiency, Efficiency of the flexibility subtest.

### 3.2 Reliability

The AREF’s reliability of each subtest was measured by Cronbach’s alpha.

Regarding Graphophonological-Semantic Flexibility task, its internal consistency was low (0.566), as indicated in Table 15.

**Table 15:** Reliability statistics of the graphophonological-semantic flexibility (GSF) subtest.

Estimate	Cronbach’s α
Point estimate	0.566
95% CI lower bound	0.502
95% CI upper bound	0.630

CI, Confidence interval.

Item-rest correlation of Graphophonological-Semantic Flexibility subtest is presented in Table 16. The points obtained by appropriate locations in the matrix as well as those obtained by correct justifying had a weak positive correlation with the total score on the other items. The time measures, on the other hand, exhibited negative correlations with the total score ranging from weak to moderate.

**Table 16:** Reliability statistics of the items in the graphophonological-semantic flexibility (GSF) subtest.

Item	Item-rest correlation
Matrix 1 - Points	−0.231
Matrix 1 – Justification	−0.240
Matrix 1 – Time	0.486
Matrix 2 – Points	−0.295
Matrix 2 – Justification	−0.095
Matrix 2 – Time	0.740
Matrix 3 – Points	−0.266
Matrix 3 – Justification	−0.185
Matrix 3 - Time	0.630

Concerning Inhibitory Control and Flexibility subtestes, their internal consistency was acceptable (0.768), as shown by Table 17.

**Table 17:** Reliability statistics of the Inhibitory Control and Flexibility subtests.

Estimate	Cronbach’s α
Point estimate	0.768
95% CI lower bound	0.753
95% CI upper bound	0.786

CI, Confidence interval.

In Table 18 are indicated item-rest correlation of Inhibitory Control and Flexibility subtests. The correlations of punctuations obtained by story retelling and question answering with the total score were positive, ranging from weak to moderate. In relation to reading times, their correlations with total score were negative, ranging from moderate to high.

**Table 18:** Reliability statistics of the items in the Inhibitory Control and Flexibility subtests.

Item	Item-rest correlation
BT - Reading time	0.924
BT - Retelling	−0.361
BT - Questions	−0.290
IC-1 - Reading time	0.945
IC-1 - Retelling	−0.161
IC-1 - Questions	−0.165
IC-2 - Reading time	0.942
IC-2 - Retelling	−0.077
IC-2 - Questions	−0.344
FL - Reading time	0.777
FL - Retelling	−0.075
FL - Questions	−0.074

Regarding the Working Memory subtest, Cronbach’s Alpha showed a high internal consistency (0.881), as illustrated in Table 19.

**Table 19:** Reliability statistics of the working memory subtest.

Estimate	Cronbach’s α
Point estimate	0.881
95% CI lower bound	0.848
95% CI upper bound	0.909

CI, Confidence interval.

The item-rest correlation of Working Memory subtest is reported in Table 20. It revealed positive correlations with total score, ranging from moderate to high.

**Table 20:** Reliability statistics of the items in the working memory subtest.

Item	Item-rest correlation
WM1	0.493
WM2	0.678
WM3	0.704
WM4	0.661
WM5	0.724
WM6	0.738
WM7	0.840

## 4 Discussion

The primary goal of this article was to furnish evidence regarding the construct validity and reliability of a new neuropsychological test designed to evaluate both executive functions and reading comprehension. Convergent validity was indicated by correlation results, concurrent validity was verified by the prediction of outcomes at the time of task performance (school year and type of school) and reliability was measured by internal consistency.

The results evidenced satisfactory psychometric qualities of the constructed tasks, manifested by significant and positive correlations with external measures of executive functions and reading comprehension, as well as adequate internal consistency of the AREF tasks. The GSF subtest showed expected correlations with reading measures, executive functions, and the Verbal IQ T-score of the WASI verbal IQ task. Although these correlations were weak, they are aligned with initial expectations, suggesting that graphophonological-semantic flexibility may serve as a relevant indicator of reading comprehension, corroborating previous findings by Cartwright (2007), Cartwright et al. (2010), and Varghese and Shanbal (2024). Additionally, it was observed that the inhibition measure of the FDT test correlated significantly with the GSF task, while the flexibility measure did not show correlation. This result can be interpreted in light of previous studies indicating that inhibition is a process that precedes flexibility (Diamond, 2013).

In the two Inhibitory Control subtests, significant correlations were identified with the reading comprehension measures (PROLEC and PROLEC-SE-R), inhibition percentile obtained in the FDT and the verbal IQ measure. The convergence between the results of the AREF Inhibitory Control tasks and the external reading measures indicate that the two share the same required construct, namely reading comprehension. The correlations between the results of the AREF Inhibitory Control subtests and the inhibition measure of the FDT align with our initial hypothesis that these relationships would be significant and positive. This finding reinforces the construct validity of the instrument, considering that the FDT demonstrates correlations with inhibitory control measures (De Paula et al., 2017). Previous studies also corroborated a higher correlation between reading comprehension tasks and Verbal IQ compared to Performance IQ (López-Escribano et al., 2013; Ready et al., 2013).

Similarly to those AREF subtests, the Flexibility subtest demonstrated positive correlations with PROLEC and PROLEC-SE-R results, with the inhibition percentile of the FDT and the verbal IQ measure as well. However, like the GSF task, the Flexibility subtest showed correlation only with the inhibitory control measure, not demonstrating correlation with the flexibility measure. One possible explanation for this finding is that, despite the task being initially developed to measure flexibility, it may recruit more inhibition processes. It is noteworthy, however, the previously mentioned observation that inhibition precedes flexibility (Diamond, 2013). Therefore, these initial findings may suggest the recruitment of this process in the task, something that should be investigated in future studies with larger samples.

In relation to inhibitory control tasks, our initial hypothesis was that an increase in distractors would lead to reduced reading efficiency compared to the performance observed in the Baseline Text. Indeed, the study by Borella and De Ribaupierre (2014) identified that resistance to distractors, measured through an external task to reading assessment (Color Stroop Task), was one of the predictors of text comprehension. However, the analyses conducted with the two inhibitory control tasks comprising the AREF resemble more closely those conducted in studies where distractors were part of the text read by participants (Connelly et al., 1991; Kemper and McDowd, 2006), and the presence of these elements was associated with reduced reader performance. Similarly to these previous studies, in the current research, participant performance was significantly lower when distractors were present in the text, albeit this was observed only in the second inhibitory control task (IC-2). In the context of the AREF tasks, the significant difference observed in the comparison between Baseline Text and IC-2 can be explained by the presence of two distractor stimuli (colored lines and target color words that should not be read) in the latter. The inclusion of more distractors may have increased the cognitive demand of the task, possibly resulting in lower average performance. Performance on the Flexibility task did not show significant differences compared to the Baseline Text. Although previous studies have shown unique contributions of flexibility to reading comprehension (Colé et al., 2014; Hung and Loh, 2021), as far as we know, no research has investigated cognitive flexibility during the reading of a text that required response alternation, such as the AREF task. Our initial hypothesis was that the demand of the flexibility task would reduce its efficiency, but this hypothesis was not confirmed. Therefore, further investigation with a larger sample is needed to confirm the consistency of the results of the inhibitory control and flexibility tasks.

In the Working Memory (WM) subtest, significant, positive and moderate correlations were observed between task results and external measures, such as reading subtests from PROLEC and PROLEC-SE-R, digit span in forward order, Scaled Scores of the WISC-IV Digit Span task, and the T-score of the Vocabulary subtest of the WASI. It is noteworthy that the latter correlation proved to be more robust than that observed between the AREF subtests and the T-score of the WASI Matrix Reasoning subtest, as predicted in the hypotheses formulated. These results not only provide support for external construct validity but also corroborate previous conclusions. For example, this is consistent with evidence that vocabulary and working memory are predictive of reading performance in children, as highlighted by Piccolo and Salles (2013). Another study (Babayiğit, 2015) indicated that differences in reading comprehension performance between individuals who had English as their first language (L1) and those who had English as their second language (L2) were explained by differences in oral language skills in English (including vocabulary and verbal working memory), with higher scores in the L1 group in both textual comprehension and oral language skills. Longitudinal data (Holahan et al., 2018), following students from grades 1 to 9, also found unique contributions of vocabulary to the development of reading comprehension. Additionally, as emphasized in the review by Butterfuss and Kendeou (2018), working memory plays an essential role in reading comprehension, as the central executive component facilitates restricting information in the phonological loop, especially in contexts where sentences become longer and syntactically more complex. This observation is consistent with the results of this study, in which the Working Memory task demands greater use of working memory as texts become longer. Nevertheless, the correlation between the AREF Working Memory result and backward digit span did not reach significance, contrary to our initial hypothesis.

The data from the present study indicated variations in the performance of the AREF subtests among participants from different school grades and between those from public and private schools. These results support the concurrent validity of the tool.

First, it was found that, in all subtests, there were statistically significant differences in student performance, with the 4th and 5th-grade results being notably lower than those of other grades in most comparisons. These findings are consistent with developmental literature, which reports cognitive improvements in the age range covered by this study, both in terms of executive functions (Jacobsen et al., 2017) and reading comprehension (De Oliveira et al., 2023). It should be noted, however, that this effect may also be associated with the presence of only public school students in the 4th and 5th-grade sample. Future studies should include younger students from private schools to verify if this result remains robust.

To prevent the average performance of public school participants from being lowered due to the inclusion of younger grades, the analyses comparing the performance of individuals from public and private schools on AREF subtests were conducted only with students from the 6th to 9th grades, as these groups included students from both types of schools. The results revealed higher average scores among private school students in WM, IC-1, and FL tasks, with no significant differences in GSF and IC-2 tasks. Although the present study did not collect data on participants’ socioeconomic status, the differences between the two groups may be related to this factor, as found in other studies (Cáceres-Serrano and Alvarado-Izquierdo, 2017; Çigdemir and Akyol, 2022; Jacobsen et al., 2017).

This study also presented indications of reliability of the AREF instrument. Regarding internal consistency, the Graphophonological-Semantic Flexibility subtest showed low levels of consistency, possibly due to their multidimensionality and the sample size (Cortina, 1993). The hypothesis of multidimensionality can be raised because the items comprising that subtest involve both scores related to the correct task performance and time measures.

On the other hand, in relation to Inhibitory Control and Flexibility, Cronbach’s alpha coefficient indicated moderate internal consistency, while the item-total correlation revealed that performance on specific items correlated weakly to strongly with total task performance. Notably, the most strongly correlated items with overall task performance were those related to timing measures, indicating that shorter reading periods were associated with better performance in the AREF. The same result was observed in the Graphophonological-Semantic Flexibility subtest, where the score on the scale was negatively related to the time spent on its completion. These observations are in line with evidence suggesting a negative relationship between accuracy in executive function tests and execution time (Camerota et al., 2019). Similarly, reduced reading speed is related to overload in working memory, resulting in reduced availability of attentional resources for reading comprehension (Zoccolotti et al., 2016; Rispens, 2004). Therefore, regarding the assessment of the two main constructs measured by the AREF - Reading Comprehension and Executive Functions -, the data suggest that longer task completion times are associated with inferior performance, which was supported by this study.

In contrast, the internal consistency of the Working Memory task was considered high, with items showing strong positive correlations with overall task performance. It indicates good reliability of this task.

The results of this study corroborate previous findings highlighting the interdependence of executive functions, such as inhibitory control, cognitive flexibility and working memory, with reading skills. However, it is crucial to interpret these results in light of the study’s limitations. Firstly, it is important to note that the research did not include a sample from the private school population of 4th and 5th grade elementary school students. Another relevant limitation is the composition of the recruited participants. Although there was a variety of age ranges, covering students aged 8 to 14 years, the study had a relatively small sample of students. Additionally, the research focused exclusively on students from the southeastern region of Brazil, which may limit the generalization of the results to the overall population. Lastly, another limitation of this study is the lack of socioeconomic data that could have been included in the statistical analyses. The inclusion of these data could be important for interpreting the results, especially considering that socioeconomic factors have shown significant correlations with both vocabulary and reading comprehension development (Lervåg et al., 2019; Olsen and Huang, 2022) as well as executive functions (Last et al., 2018; Lawson et al., 2018).

Based on the results obtained, it is possible to conclude that the AREF instrument presents initial psychometric evidence indicating its viability for clinical and research use after obtaining a normative sample. Although the strengths of the correlations with other instruments range from weak to moderate, this can be attributed to the many factors influencing performance on the complex target constructs: reading comprehension and executive functions. Considering the complexity of evaluating both constructs, it is a significant achievement that the test has demonstrated construct validity evidence for both variables, indicating its utility, especially in the Brazilian context, where no equivalent exists.

However, it is evident that further studies are necessary to reinforce the psychometric validation of the developed subtests. Specifically, the lack of correlation of the Flexibility task and Graphophonological-Semantic Flexibility of the AREF with external measures of flexibility highlights the need for a more in-depth investigation to determine if the subtests are truly assessing what it intends to. Additionally, for the IC/FL and GSF subtests, it would be important to conduct further reliability analysis using methods more sensitive to the multidimensionality of the tasks. Without these analyses, the scores obtained by individuals undergoing the application should be interpreted with caution. Regarding the working memory subtest, where time is not a variable, the measure of external consistency was high, and the correlations with external measures support its construct validity, suggesting it is suitable for use.

Future studies with larger and more representative samples are essential to replicate the findings obtained and determine if these findings can be extrapolated to other populations. Additionally, it is crucial to conduct further research to evaluate the instrument’s sensitivity regarding students reporting difficulties in reading comprehension and executive deficits, both in the presence and absence of mental disorders. Furthermore, the importance of conducting normative studies to establish parameters that allow for the interpretation of data obtained with students and patients is emphasized.

Despite significant challenges associated with creating tasks capable of simultaneously assessing reading processes and executive functions, the findings of this study suggest that the AREF appears to fulfill this complex purpose effectively. This finding has promising implications, indicating that the AREF may be a useful tool in the neuropsychological assessment of children and adolescents with reading comprehension difficulties, as well as in cases of isolated executive dysfunctions or as part of various neurodevelopmental disorders, including specific learning disorders and attention deficit hyperactivity disorder (ADHD).

Furthermore, the data obtained through the AREF have the potential to support the planning of therapeutic interventions in various areas, including neuropsychology, speech therapy, and educational psychology. A deeper understanding of the performance patterns of these individuals will allow for a more personalized approach to help them overcome their specific difficulties.

## Data Availability

The original statistical analysis presented in the study are included in the article/Supplementary material, further inquiries can be directed to the corresponding author.

## Supplementary material

Supplementary Figure S1 Comparison of the average performance of individuals from public and private schools on the WM task.

Supplementary Figure S2 Comparison of the average performance of individuals from public and private schools on the IC-1 task.

Supplementary Figure S3 Comparison of the average performance of individuals from public and private schools on the FL task.
