# Supplementary figures and images for: A new neuropsychological tool for simultaneous reading and executive functions assessment: initial psychometric properties
Source: Front Psychol. 2024 Sep 23;15:1399388. doi: 10.3389/fpsyg.2024.1399388 (PMC11456448; doi:10.3389/fpsyg.2024.1399388)

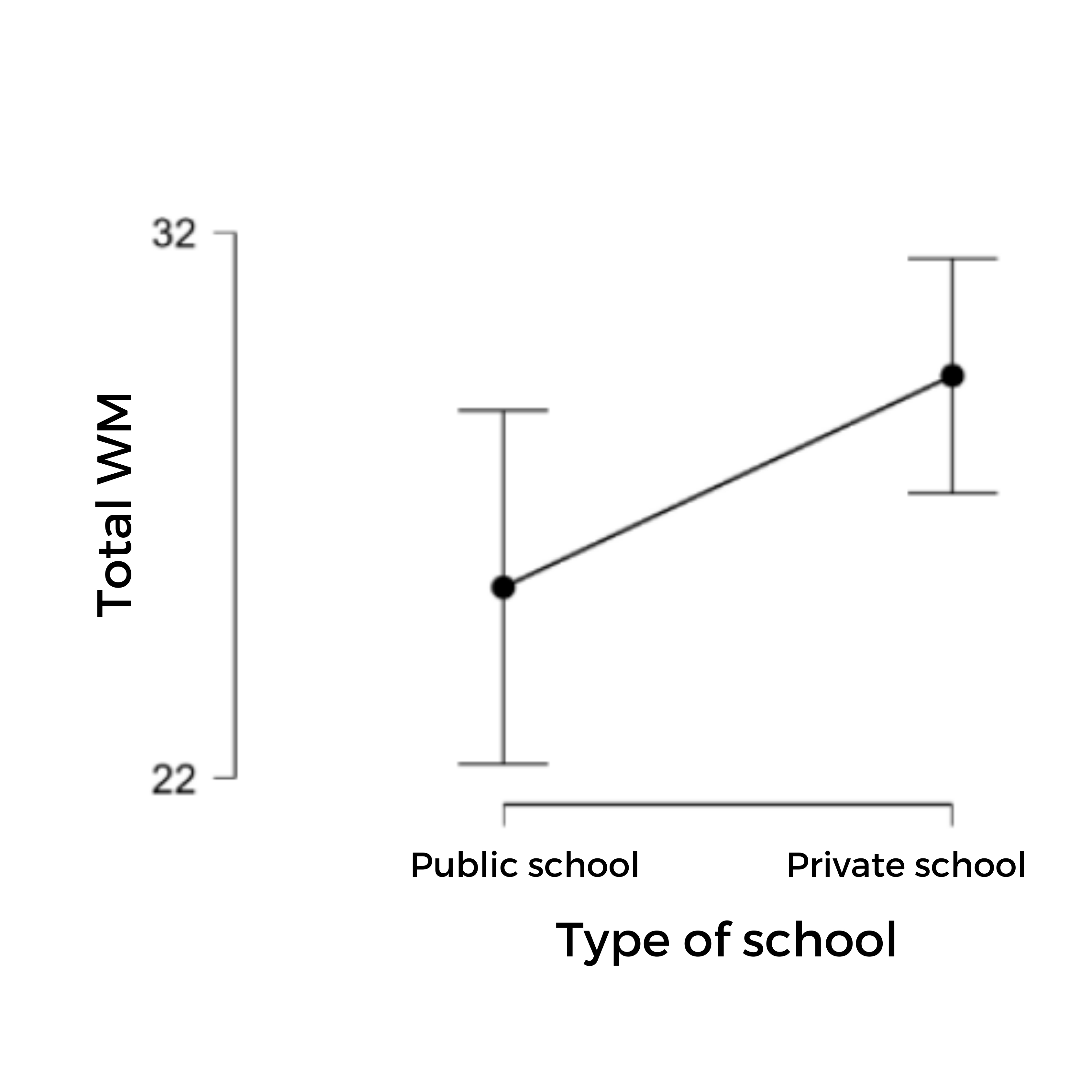

Supplement: Supplementary Figure S1 — Comparison of the average performance of individuals from public and private schools on the WM task. [file Image_1.jpg]

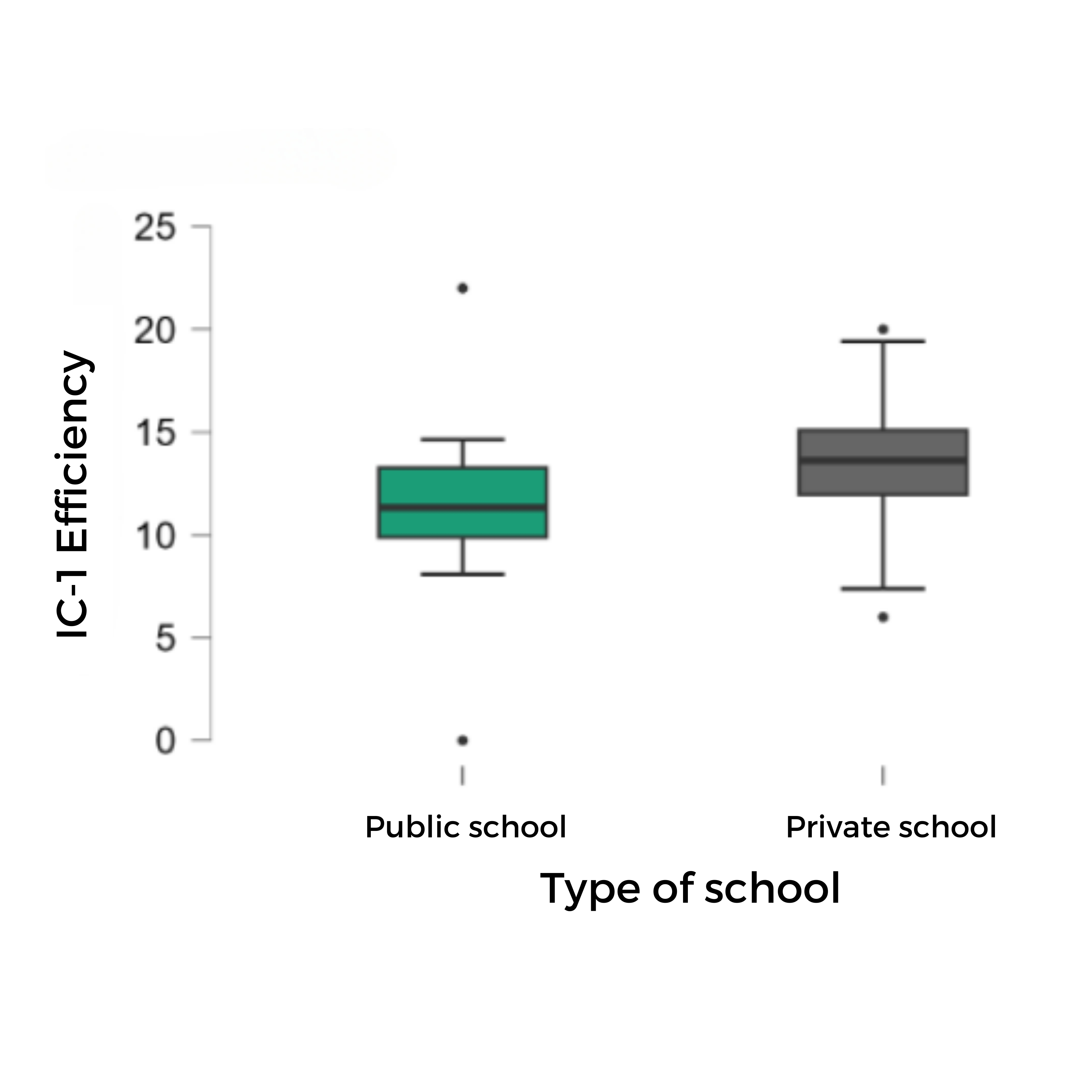

Supplement: Supplementary Figure S2 — Comparison of the average performance of individuals from public and private schools on the IC-1 task. [file Image_2.jpg]

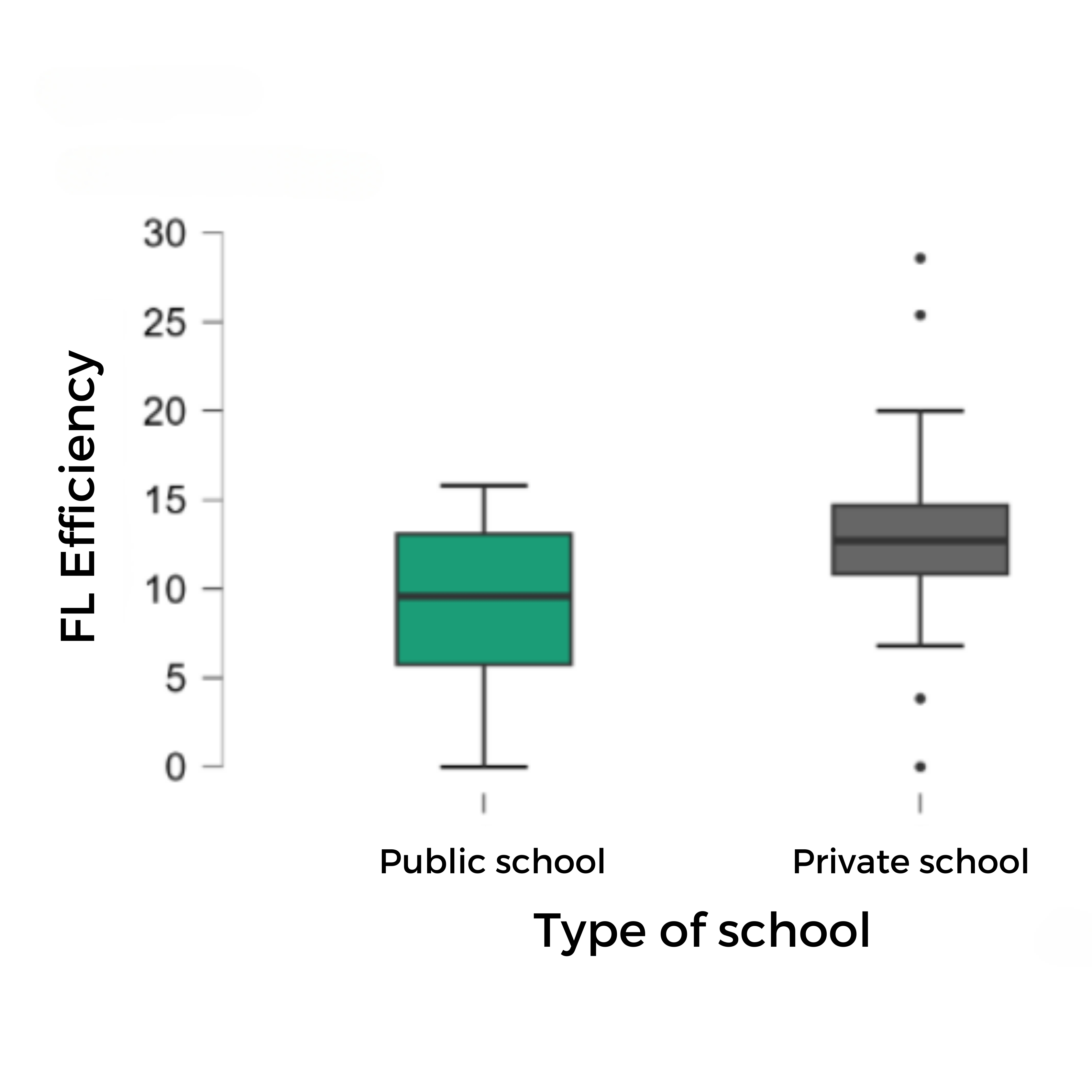

Supplement: Supplementary Figure S3 — Comparison of the average performance of individuals from public and private schools on the FL task. [file Image_3.jpg]
