# Supplementary material for: A new neuropsychological tool for simultaneous reading and executive functions assessment: initial psychometric properties
Source: Front Psychol. 2024 Sep 23;15:1399388. doi: 10.3389/fpsyg.2024.1399388 (PMC11456448; doi:10.3389/fpsyg.2024.1399388)
Supplement: Supplementary file 4 [file Data_Sheet_1.pdf]

Output of Spearman correlations performed with the efficiency of the Graphophonological-Semantic Flexibility subtest of AREF. From JASP software.

Results

Correlation

Spearman's Correlations

Variable		GSF-Ef	Inhibition - PC	Flexibility - PC	PROLEC Classification	WASI Vocabulary	WASI Matrix reasoning
1. GSF-Ef	Spearman's rho	—					
	p-value	—					
2. Inhibition - PC	Spearman's rho	0.209*	—				
	p-value	0.045	—				
3. Flexibility - PC	Spearman's rho	0.116	0.622***	—			
	p-value	0.266	< .001	—			
4. PROLEC Classification	Spearman's rho	0.355***	0.109	0.101	—		
	p-value	< .001	0.298	0.335	—		
5. WASI Vocabulary	Spearman's rho	0.348***	0.174	0.221*	0.256*	—	
	p-value	< .001	0.096	0.033	0.013	—	
6. WASI Matrix reasoning	Spearman's rho	0.109	0.274**	0.319**	0.038	0.189	—
	p-value	0.298	0.008	0.002	0.718	0.070	—

* p < .05, ** p < .01, *** p < .001

Output of Spearman correlations performed with the efficiency of the Baseline Text, the Inhibitory Control and the Flexibility subtests of AREF. From JASP software.

Correlation

Spearman's Correlations

Variable		BT-Ef	IC1-Ef	IC2-Ef	FL-Ef	Inhibition - PC	Flexibility - PC	PROLEC Classification	WASI Vocabulary	WASI Matrix reasoning
1. BT-Ef	Spearman's rho	—								
	p-value	—								
2. IC1-Ef	Spearman's rho	0.767***	—							
	p-value	< .001	—							
3. IC2-Ef	Spearman's rho	0.731***	0.801***	—						
	p-value	< .001	< .001	—						
4. FL-Ef	Spearman's rho	0.710***	0.691***	0.627***	—					
	p-value	< .001	< .001	< .001	—					
5. Inhibition - PC	Spearman's rho	0.300**	0.387***	0.284**	0.358***	—				
	p-value	0.004	< .001	0.006	< .001	—				
6. Flexibility - PC	Spearman's rho	0.112	0.265*	0.203	0.100	0.622***	—			
	p-value	0.286	0.010	0.051	0.341	< .001	—			
7. PROLEC Classification	Spearman's rho	0.339***	0.367***	0.339***	0.358***	0.109	0.101	—		
	p-value	< .001	< .001	< .001	< .001	0.298	0.335	—		
8. WASI Vocabulary	Spearman's rho	0.307**	0.412***	0.390***	0.262*	0.174	0.221*	0.256*	—	
	p-value	0.003	< .001	< .001	0.011	0.096	0.033	0.013	—	
9. WASI Matrix reasoning	Spearman's rho	0.131	0.172	0.124	0.185	0.274**	0.319**	0.038	0.189	—
	p-value	0.211	0.099	0.236	0.076	0.008	0.002	0.718	0.070	—

* p < .05, ** p < .01, *** p < .001

Output of Spearman correlations performed with the Working Memory subtest of AREF . From JASP software. From JASP software.

Correlation

Spearman's Correlations

Variable		WM TOTAL	Digit Span - SS	Forward Span	Backward Span	PROLEC Classification	WASI Vocabulary	WASI Matrix reasoning
1. WM TOTAL	Spearman's rho	—						
	p-value	—						
2. Digit Span - SS	Spearman's rho	0.259*	—					
	p-value	0.012	—					
3. Forward Span	Spearman's rho	0.396***	0.746***	—				
	p-value	< .001	< .001	—				
4. Backward Span	Spearman's rho	0.160	0.665***	0.434***	—			
	p-value	0.125	< .001	< .001	—			
5. PROLEC Classification	Spearman's rho	0.365***	0.135	0.150	0.081	—		
	p-value	< .001	0.197	0.151	0.438	—		
6. WASI Vocabulary	Spearman's rho	0.328**	0.132	0.214*	0.012	0.256*	—	
	p-value	0.001	0.206	0.040	0.909	0.013	—	
7. WASI Matrix reasoning	Spearman's rho	0.241*	0.275**	0.105	0.106	0.038	0.189	—
	p-value	0.020	0.008	0.318	0.312	0.718	0.070	—

* p < .05, ** p < .01, *** p < .001

Output from the Cronbach's Alpha analysis of the Graphophonological-Semantic Flexibility subtest. From JASP software.

Unidimensional Reliability ▼

Frequentist Scale Reliability Statistics

Estimate	Cronbach's α
Point estimate	0.566
95% CI lower bound	0.503
95% CI upper bound	0.629

Note. Of the observations, pairwise complete cases were used. The following items correlated negatively with the scale: Matrix 1 - Time, Matrix 2 - Time, Matrix 3 - Time.

Frequentist Individual Item Reliability Statistics ▼

Item	Item-rest correlation
Matrix 1 - Points	−0.231
Matrix 1 - Justification	−0.240
Matrix 1 - Time	0.486
Matrix 2 - Points	−0.295
Matrix 2 - Justification	−0.095
Matrix 2 - Time	0.740
Matrix 3 - Points	−0.266
Matrix 3 - Justification	−0.185
Matrix 3 - Time	0.630

Output from the Cronbach's Alpha analysis of the Inhibitory Control and Flexibility Subtests. From JASP software.

Unidimensional Reliability ▼

Frequentist Scale Reliability Statistics

Estimate	Cronbach's α
Point estimate	0.768
95% CI lower bound	0.753
95% CI upper bound	0.786

Note. Of the observations, pairwise complete cases were used. The following items correlated negatively with the scale: BT - Reading time, IC-1 - Reading time, IC-2 - Reading time3, FL - Reading time.

Frequentist Individual Item Reliability Statistics ▼

Item	Item-rest correlation
BT - Reading time	0.924
BT- Retelling	−0.361
BT - Questions	−0.290
IC-1 - Reading time	0.945
IC-1 - Retelling	−0.161
IC-1 - Questions	−0.165
IC-2 - Reading time3	0.942
IC-2 - Retelling	−0.077
IC-2 - Questions	−0.344
FL - Reading time	0.777
FL - Retelling	−0.075
FL - Questions	−0.074

Output from the Cronbach's Alpha analysis of the Working Memory Subtest. From JASP software.

Unidimensional Reliability ▼

Frequentist Scale Reliability Statistics

Estimate	Cronbach's α
Point estimate	0.881
95% CI lower bound	0.848
95% CI upper bound	0.909

Note. Of the observations, pairwise complete cases were used.

Frequentist Individual Item Reliability Statistics ▼

Item	Item-rest correlation
WM1	0.493
WM2	0.678
WM3	0.704
WM4	0.661
WM5	0.724
WM6	0.738
WM7	0.840

Output of ANOVA conducted on the efficiency scores of the Graphophonological-Semantic Flexibility subtest of AREF, comparing students from grades 4 to 9. From JASP software.

ANOVA - Eficiência - Matrizes

Cases	Sum of Squares	df	Mean Square	F	p	η^2
Ano	65.670	5	13.134	8.115	< .001	0.318
Residuals	140.801	87	1.618			

Note. Type III Sum of Squares

Post Hoc Tests ▼

Standard ▼

Post Hoc Comparisons - Ano ▼

		Mean Difference	SE	t	p_{tukey}
4	5	0.084	0.397	0.211	1.000
	6	−1.553	0.451	−3.447	0.011
	7	−1.285	0.443	−2.900	0.052
	8	−1.918	0.469	−4.092	0.001
	9	−1.804	0.604	−2.986	0.041
5	6	−1.637	0.412	−3.970	0.002
	7	−1.369	0.404	−3.387	0.013
	8	−2.002	0.432	−4.633	< .001
	9	−1.888	0.576	−3.276	0.018
6	7	0.268	0.457	0.587	0.992
	8	−0.365	0.482	−0.756	0.974
	9	−0.250	0.615	−0.407	0.999
7	8	−0.633	0.475	−1.333	0.766
	9	−0.519	0.609	−0.852	0.957
8	9	0.114	0.628	0.182	1.000

Note. P-value adjusted for comparing a family of 6

Output of ANOVA conducted on the efficiency scores of the first task of the Inhibitory Control subtest of AREF, comparing students from grades 4 to 9. From JASP software.

ANOVA - Eficiência - Texto 2

Cases	Sum of Squares	df	Mean Square	F	p	η^2
Ano	777.688	5	155.538	10.898	< .001	0.385
Residuals	1241.629	87	14.272			

Note. Type III Sum of Squares

Post Hoc Tests ▼

Standard ▼

Post Hoc Comparisons - Ano ▼

		Mean Difference	SE	t	p_{tukey}
4	5	−0.185	1.178	−0.157	1.000
	6	−5.396	1.338	−4.032	0.002
	7	−5.338	1.316	−4.057	0.001
	8	−6.133	1.392	−4.406	< .001
	9	−7.480	1.794	−4.170	< .001
5	6	−5.212	1.225	−4.255	< .001
	7	−5.153	1.200	−4.293	< .001
	8	−5.948	1.283	−4.635	< .001
	9	−7.295	1.711	−4.264	< .001
6	7	0.058	1.358	0.043	1.000
	8	−0.737	1.432	−0.515	0.995
	9	−2.084	1.825	−1.142	0.862
7	8	−0.795	1.411	−0.564	0.993
	9	−2.142	1.808	−1.184	0.843
8	9	−1.347	1.865	−0.722	0.979

Note. P-value adjusted for comparing a family of 6

Output of ANOVA conducted on the efficiency scores of the second task of the Inhibitory Control subtest of AREF, comparing students from grades 4 to 9. From JASP software.

ANOVA - Eficiência - Texto 3

Cases	Sum of Squares	df	Mean Square	F	p	η²
Ano	977.421	5	195.484	7.888	< .001	0.312
Residuals	2155.993	87	24.782			

Note. Type III Sum of Squares

Post Hoc Tests ▼

Standard ▼

Post Hoc Comparisons - Ano ▼

		Mean Difference	SE	t	p_{tukey}
4	5	−1.229	1.553	−0.791	0.968
	6	−5.325	1.763	−3.020	0.038
	7	−7.380	1.734	−4.256	< .001
	8	−7.964	1.834	−4.342	< .001
	9	−7.941	2.364	−3.359	0.014
5	6	−4.096	1.614	−2.538	0.125
	7	−6.151	1.582	−3.889	0.003
	8	−6.736	1.691	−3.983	0.002
	9	−6.713	2.255	−2.977	0.042
6	7	−2.055	1.789	−1.149	0.859
	8	−2.640	1.886	−1.399	0.727
	9	−2.616	2.405	−1.088	0.885
7	8	−0.585	1.859	−0.315	1.000
	9	−0.561	2.383	−0.236	1.000
8	9	0.023	2.457	0.009	1.000

Note. P-value adjusted for comparing a family of 6

Output of ANOVA conducted on the efficiency scores of the Flexibility subtest of AREF, comparing students from grades 4 to 9. From JASP software.

ANOVA - Eficiência - Texto 4

Cases	Sum of Squares	df	Mean Square	F	p	η²
Ano	601.656	5	120.331	5.540	< .001	0.242
Residuals	1889.571	87	21.719			

Note. Type III Sum of Squares

Post Hoc Tests

Standard

Post Hoc Comparisons - Ano

		Mean Difference	SE	t	p_{tukey}
4	5	−0.058	1.454	−0.040	1.000
	6	−3.532	1.651	−2.139	0.277
	7	−5.172	1.623	−3.186	0.024
	8	−5.404	1.717	−3.147	0.027
	9	−6.639	2.213	−3.000	0.040
5	6	−3.475	1.511	−2.299	0.206
	7	−5.115	1.481	−3.454	0.011
	8	−5.347	1.583	−3.377	0.014
	9	−6.582	2.111	−3.118	0.029
6	7	−1.640	1.675	−0.979	0.923
	8	−1.872	1.766	−1.060	0.896
	9	−3.107	2.251	−1.380	0.739
7	8	−0.232	1.740	−0.133	1.000
	9	−1.467	2.231	−0.657	0.986
8	9	−1.235	2.300	−0.537	0.994

Note. P-value adjusted for comparing a family of 6

Output of ANOVA conducted on the total score from Working Memory subtest of AREF, comparing students from grades 4 to 9. From JASP software.

ANOVA - Total MO

Cases	Sum of Squares	df	Mean Square	F	p	η²
Ano	2505.078	5	501.016	10.345	< .001	0.373
Residuals	4213.653	87	48.433			

Note. Type III Sum of Squares

Post Hoc Tests ▼

Standard

Post Hoc Comparisons - Ano

		Mean Difference	SE	t	p_{tukey}
4	5	−1.152	2.171	−0.531	0.995
	6	−8.749	2.465	−3.549	0.008
	7	−11.570	2.424	−4.773	< .001
	8	−11.575	2.564	−4.514	< .001
	9	−12.716	3.305	−3.848	0.003
5	6	−7.597	2.256	−3.367	0.014
	7	−10.418	2.211	−4.711	< .001
	8	−10.423	2.364	−4.409	< .001
	9	−11.564	3.152	−3.669	0.005
6	7	−2.821	2.501	−1.128	0.869
	8	−2.826	2.637	−1.071	0.891
	9	−3.967	3.362	−1.180	0.845
7	8	−0.005	2.599	−0.002	1.000
	9	−1.146	3.332	−0.344	0.999
8	9	−1.141	3.435	−0.332	0.999

Note. P-value adjusted for comparing a family of 6

Output of the comparison between the performance of students from public and private schools on the Graphophonological-Semantic Flexibility subtest of AREF, comparing students from grades 6th to 9th. Analysis performed using JASP software.
Note: 1 = Public Schools; 2 = Private Schools

Independent Samples T-Test

	t	df	p	Cohen's d	SE Cohen's d
Eficiência - Matrizes	−0.792	48	0.433	−0.233	0.297

Note. Student's t-test.

Assumption Checks ▼

Test of Normality (Shapiro-Wilk)

		W	p
Eficiência - Matrizes	1	0.974	0.869
	2	0.964	0.356

Note. Significant results suggest a deviation from normality.

Test of Equality of Variances (Levene's)

	F	df_1	df_2	p
Eficiência - Matrizes	0.447	1	48	0.507

Descriptives

Group Descriptives

	Group	N	Mean	SD	SE	Coefficient of variation
Eficiência - Matrizes	1	18	2.748	1.314	0.310	0.478
	2	32	3.069	1.414	0.250	0.461

Output of the comparison between the performance of students from public and private schools on the first task of the Inhibitory Control subtest of AREF, comparing students from grades 6th to 9th. Analysis performed using JASP software.
Note: 1 = Public Schools; 2 = Private Schools

Independent Samples T-Test

	W	df	p	Rank-Biserial Correlation	SE Rank-Biserial Correlation
Eficiência - Texto 2	187.500		0.043	−0.349	0.170

Note. For the Mann-Whitney test, effect size is given by the rank biserial correlation.
Note. Mann-Whitney U test.

Assumption Checks ▼

Test of Normality (Shapiro-Wilk) ▼

		W	p
Eficiência - Texto 2	1	0.874	0.020
	2	0.959	0.260

Note. Significant results suggest a deviation from normality.

Descriptive Statistics

	Eficiência - Texto 2	
	1	2
Valid	18	32
Missing	0	0
Median	11.325	13.605
Std. Deviation	4.192	3.601
Minimum	0.000	6.000
Maximum	22.000	20.000

Output of the comparison between the performance of students from public and private schools on the second task of the Inhibitory Control subtest of AREF, comparing students from grades 6th to 9th. Analysis performed using JASP software.
Note: 1 = Public Schools; 2 = Private Schools

Independent Samples T-Test

	t	df	p	Cohen's d	SE Cohen's d
Eficiência - Texto 3	−1.477	48	0.146	−0.435	0.303

Note. Student's t-test.

Assumption Checks ▼

Test of Normality (Shapiro-Wilk)

		W	p
Eficiência - Texto 3	1	0.972	0.833
	2	0.982	0.861

Note. Significant results suggest a deviation from normality.

Test of Equality of Variances (Levene's)

	F	df_1	df_2	p
Eficiência - Texto 3	1.282	1	48	0.263

Descriptives

Group Descriptives

	Group	N	Mean	SD	SE	Coefficient of variation
Eficiência - Texto 3	1	18	10.646	4.862	1.146	0.457
	2	32	13.043	5.830	1.031	0.447

Output of the comparison between the performance of students from public and private schools on the Flexibility subtest of AREF, comparing students from grades 6th to 9th. Analysis performed using JASP software.
Note: 1 = Public Schools; 2 = Private Schools

Independent Samples T-Test

	W	df	p	Rank-Biserial Correlation	SE Rank-Biserial Correlation
Eficiência - Texto 4	190.000		0.049	−0.340	0.170

Note. For the Mann-Whitney test, effect size is given by the rank biserial correlation.
Note. Mann-Whitney U test.

Assumption Checks ▼

Test of Normality (Shapiro-Wilk) ▼

		W	p
Eficiência - Texto 4	1	0.945	0.358
	2	0.926	0.031

Note. Significant results suggest a deviation from normality.

Descriptive Statistics

	Eficiência - Texto 4	
	1	2
Valid	18	32
Missing	0	0
Median	9.575	12.700
Std. Deviation	4.841	5.381
Minimum	0.000	0.000
Maximum	15.790	28.570

Output of the comparison between the performance of students from public and private schools on the Working Memory subtest of AREF, comparing students from grades 6th to 9th. Analysis performed using JASP software.
Note: 1 = Public Schools; 2 = Private Schools

Independent Samples T-Test

	t	df	p	Cohen's d	SE Cohen's d
Total MO	−2.135	48	0.038	−0.629	0.313

Note. Student's t-test.

Assumption Checks ▼

Test of Normality (Shapiro-Wilk) ▼

		W	p
Total MO	1	0.964	0.681
	2	0.971	0.522

Note. Significant results suggest a deviation from normality.

Test of Equality of Variances (Levene's)

	F	df_1	df_2	p
Total MO	0.014	1	48	0.906

Group Descriptives

	Group	N	Mean	SD	SE	Coefficient of variation
Total MO	1	18	25.500	6.510	1.534	0.255
	2	32	29.375	5.961	1.054	0.203
